# Frequency of peripheral blood eosinophilia and obstructive airway disease in sarcoidosis

**DOI:** 10.3906/sag-2007-87

**Published:** 2021-11-04

**Authors:** Bilal RABAHOĞLU, Fatma Sema OYMAK, Burcu BARAN KETENCİOĞLU, Nuri TUTAR, İnci GÜLMEZ, İnsu YILMAZ

**Affiliations:** 1 Department of Pulmonary Medicine, School of Medicine, Erciyes University, Kayseri, Turkey; 2 Division of Immunology and Allergy, Department of Pulmonary Medicine, School of Medicine, Erciyes University, Kayseri, Turkey

**Keywords:** Airway obstruction, asthma, eosinophilia, sarcoidosis

## Abstract

**Background/aim:**

There is limited information about peripheral blood eosinophilia (PBE) and airway obstruction in sarcoidosis. Since pulmonary sarcoidosis affects the airways, it is often confused with asthma. The aims of the study are to investigate airway obstruction and PBE in sarcoidosis patients and to examine the similarity of clinical presentation with asthma.

**Materials and methods:**

The patients matching the ATS/ERS/WASOG diagnosis criteria and were between 18 and 80 years of age were included consecutively between 2018 and 2020. Other diseases causing granulomas were excluded.

**Results:**

A total of 84 patients were included of which 26 (31%) had a PBE level of ≥300 μL with no significant difference seen between sarcoidosis stage and PBE (p > 0.05). A significant (p < 0.05) decrease was only seen in FEV1 as the stage of sarcoidosis progressed. Respectively 31 (36.9%), 12 (14.3%) and 4 (4.8%) patients had an obstructive, restrictive and mixed respiratory function disorder. Twenty-four (28.6%) subjects with sarcoidosis had history of asthma. Spring fever, eczema, and skin/nose allergy were noticed in 17 (20.2%) of the patients.

**Conclusion:**

Mild PBE may be seen in sarcoidosis. Patients applying with PBE, airway obstruction, bronchial hyperreactivity along with spring fever, eczema, skin/nose allergy, wheezing, chest tightness, shortness of breath and cough may be also evaluated in terms of sarcoidosis.

## 1. Introduction

Sarcoidosis is a systemic disease which causes an immune-related noncaseous granuloma formation in the involved organ [1]. This disease can be seen worldwide and affects all ages and races [2]. Genetic, infectious and environmental factors along with occupational exposures are thought to be etiologic factors of sarcoidosis [3,4].

Although most patients with sarcoidosis have normal pulmonary function test (PFT), restrictive, obstructive or mixed disorder can also be observed [5]. Airway involvement is common in sarcoidosis and granulomatous inflammation can be seen in approximately 60% of endobrochial biopsies [6]. Endobronchial granulomas may cause narrowing, distortion or complete obstruction in the airways [7,8]. Sarcoid granulomas can also progress to fibrosis and cause severe contraction and distortion in the airways [9,10]. Temporary airway obstruction can be seen in up to 58% of sarcoidosis patients due to bronchial hyperreactivity and bronchospasm [11–13]. Restrictive disorders usually result from advanced parenchymal fibrosis due to interstitial pneumonia which is accompanied by sarcoid granulomas and/or sarcoidosis. Since sarcoidosis affects lung structure, it is generally characterized as a restrictive lung disease and characterized by low lung compliance [14]. Diffusing capacity of carbon monoxide (DLCO) is the most common PFT disorder in sarcoidosis and the most sensitive test for functional alveolar surface loss; a decrease in DLCO is thought to be due to the presence of parenchymal involvement and/ or pulmonary hypertension. Hemoglobin concentration also plays an important role in DLCO decrease. Anemia and hematological disorders are common comorbidities in sarcoidosis which are seen because of infiltration of the bone marrow with noncaseating granulomas and decreased iron storage [15]. DLCO decline is a sign of gas exchange disorder and a decrease in alveolar membrane diffusion capacity. DLCO drop and desaturation during exercise is the strongest functional parameter to demonstrate the severity and progression of sarcoidosis [16].

Eosinophilia is defined as an increase in the number of eosinophils above the normal values observed in healthy individuals [17]. No relation was observed between sex and ethnic factors in terms of eosinophil count. Eosinophilia causes are many and in general they are divided into three main categories: primary, secondary (reactive) and idiopathic [18]. In sarcoidosis patients secondary eosinophilia may be observed and according to the Guide of the British Society of Hematology, it has been explained that mild peripheral blood eosinophilia (PBE) can be seen in sarcoidosis. The frequency of mild PBE in sarcoidosis is very variable and may reach up to 25% [19].

This study aimed at evaluating airway obstruction and PBE in sarcoiosis patients. In addition, it also aimed at evaluating allergic signs/symptoms and the similarities between the clinical presentations of sarcoidosis and asthma in order to help with the differential diagnosis.

## 2. Materials and methods

The study sample consisted of 84 pulmonary sarcoidosis patients diagnosed at Erciyes University Medical Faculty. Sarcoidosis patients were recruited consecutively between 01.02.2018–01.02.2020. Prior to the study, approval was obtained from the Ethics Committee of Erciyes University Faculty of Medicine (Ethics Committee approval number: 2018/104) and the patients gave their written informed consent.

### 2.1. Patient selection

The patients were included in the study according to ATS/ERS/WASOG diagnostic criteria for sarcoidosis and were between 18 and 80 years of age. Those patients were clinically, radiologically and histopathologically (noncaseating granuloma on biopsy specimen) compatible with sarcoidosis. Other diseases causing granulomas such as typical/atypical tuberculosis, nocardia, actinomycosis, pneumocystis jirovecii infection, granulomatosis with polyangiitis, eosinophilic granulomatosis with polyangiitis and collagen tissue diseases were excluded. Diseases other than asthma causing peripheral blood eosinophilia (e.g., parasitic infections) were also excluded.

### 2.2. Staging sarcoidosis patients

Stages of sarcoidosis were determined according to the Siltzbach classification. Stage 0 was determined as normal chest X-ray, stage 1 bilateral lymphadenopathy, stage 2 bilateral lymphadenopathy with parenchymal lung disease, stage 3 only parenchymal lung disease and stage 4 pulmonary fibrosis [20].

### 2.3. Hemogram

The complete blood count (CBC) was performed as a routine test for all sarcoidosis patients applying to the outpatient clinic. The blood samples were drawn from the venous system of the forearm, collected into potasyum ethylene diamine tetra-acetate vacutainer tube and then sent to the Erciyes University biochemistry laboratory. Hemoglobin and eosinophil levels were recorded before initiation of corticosteroid treatment in patients with treatment indication. In this study, the absolute value of eosinophils 300 μL and above was accepted as PBE. The cut off value of 300 μL has been used to evaluate exacerbations and treatment indications in different disease groups [21,22].

### 2.4. Pulmonary function tests/diffusing capacity (DLCO)

Pulmonary function tests were performed with the Viasys Vmax 20c spirometer device in Erciyes University Medical Faculty Chest Diseases outpatient clinic. Tests were performed with patients in an upright sitting position. After performing at least three acceptable maneuvers for the PFT, the best test was recorded. The patients were given 400 mcg salbutamol by inhalation, and after 15 min, a respiratory function test was performed again to evaluate reversibility. Forced expiratory volume in the first second (FEV1) was evaluated before and after salbutamol inhalation and a difference of ≥200 milliliters and ≥12% in FEV1 was accepted as positive in terms of reversibility. FEV1/FVC ≤ 75% was accepted as obstructive, FEV1/FVC ≥ 75% and FVC < 80% were accepted as restrictive and FEV1/FVC ≤ 75% with FVC < 80% were accepted as mixed airway disorders.

DLCO value was measured using the Vmax Encore device and using carbon monoxide to evaluate alveolo-capillary membrane diffusion capacity corrected for the hemoglobin value of the patients. According to the measured DLCO (mL/mmHg/min) values; patients were analyzed into 5 groups as increased (>120%), normal (80%–120%), slight decrease (60%–79%), moderate decrease (40%–59%) and severe decrease (<40%) in diffusion capacity.

### 2.5. Allergy and asthma symptom survey

This survey was performed by a physician upon examination to evaluate airway obstruction, atopy, smoking, family history, self-reported asthma and asthma-like symptoms in patients with sarcoidosis [23]. This questionnaire is based on that used in the first New Zealand study [24]. It was then modified slightly from the one used in the European Community Respiratory Health Survey (ECRHS) [25]. In this study, the modified version of ECRHS was used. Before applying the survey, it was translated to the Turkish language and validated by some of the chest diseases specialists [26]. To ensure the reliability of the survey we performed it twice on the same patients 2 months apart and we got approximately the same results in both. It was depended on the history of physician diagnosed self-reported asthma for the diagnosis and on the survey questions to record the symptoms of the patients.

### 2.6. Statistics

The SPSS software version 22.0 (SSPS Inc., Chicago, Illinois, USA) was used for statistical analysis. In order to check whether the data are parametric or not, the distribution of continuous variables was tested with One-sample Kolmogorov–Smirnov and the homogenity of variances was tested with Levene test. Categorical variables were reported as frequency and group percentages. Categorical variables were analyzed with the chi-square test. In comparison of more than two groups one-way ANOVA, Scheffe test was used. All p values less than 0.05 were considered significant.

## 3. Results

Of the 84 patients enrolled in the study 14 (16.7%) were male and 70 (83.3%) were female with an average age and body mass index (BMI) of 48.39 kg/m^2^ ± 12.97 and 30.74 kg/m^2^ ± 5.26 respectively. As shown in Table 1, the average PBE was 254.04 μL ± 176.41.

### 3.1. Sarcoidosis stage and peripheral blood eosinophilia count

There were no stage 0 patients and 26 (31%), 45 (53.6%), 8 (9.5%) and 5 (6%) patients were categorized as stages 1–4, respectively. Of the 84 patients included in the study, 26 (31%) patients had an absolute PBE ≥ 300 μL on their complete blood count. Of these patients, 9 (34.6%), 12 (46.1%), 2(7.6%), and 3 (11.5%) patients had stages 1, 2, 3, and 4 sarcoidosis, respectively. There was no significant difference between the stage of sarcoidosis and PBE (p = 0.286). In this study, bronchoalveolar lavage eosinophilia was not evaluated. Out of the 26 patients with PBE, 3 were cigarette smokers while the others had no smoking history.

### 3.2. Pulmonary function test results

When evaluating the PFT of the patients according to the stage of the disease, no significant difference was observed in terms of FEV1/FVC ratio and FVC (in terms of milliliter and percentage). In contrast, a significant difference was seen in FEV1 in terms of milliliter (F = 3.842, p = 0.013) and percentage (F = 2.316, p = 0.041). The effect size (η^2^) for FEV1 (percentage) was calculated and it comes out that the effect was medium-high (η^2^ = 0.097), whereas the effect size for FEV1 (milliliter) was high (η^2^ = 0.126). As the stage progresses, FEV1 was seen to significantly decrease as shown in Table 2. As the stage of sarcoidosis progressed a significant (F = 7.278, p = 0.00) decrease was observed in DLCO. The calculated effect size for DLCO was the highest (η^2^ = 0.214).

When the pattern of respiratory dysfunction was evaluated normal, obstructive, restrictive and mixed respiratory patterns were seen in 37 (44%), 31 (36.9%), 12(14.3%), and 4 (%4.8) respectively. From the 31 patients with an obstructive PFT, 9 patients had a self-reported asthma history and 2 were smokers. Out of the 84 patients included in the study, 6 (7.1%) had a reversible obstruction in PFT, 4 of whom had a self-reported asthma history.

### 3.3. Prevalence of asthma/allergy symptoms in sarcoidosis

Twenty-four (28.6%) subjects with sarcoidosis had a self-reported history of physician diagnosed asthma and 6 (7.1%) reported an asthma attack in the last 12 months with 13 (15.5%) reporting current use of asthma medication (none of which was using oral corticosteroid) as shown in Table 3. Female patients with sarcoidosis were more likely to have a history of asthma or ever use an asthma medication than men although it was not statistically significant (p = 0.385 and 0.622, respectively). Wheezing, nocturnal symptoms and allergy were also seen more frequently in women but were not statistically significant (p = 0.484, 0.378, and 0.424, respectively). Out of the 24 self-reported asthma patients 6 had PBE and asthma diagnosis did not differ between the stages of sarcoidosis.

## 4. Discussion

Eosinophilia is described as an elevated level of eosinophils over healthy subjects. Their production takes place in the bone marrow and is tightly controlled by transcription factors and cytokines primarily Interleukin IL-5, IL-3 and granulocyte-macrophage colony-stimulating factor. These cytokines are the principle drivers of reactive (secondary) eosinophilia which is seen in sarcoidosis and many other lung diseases [18]. In this study, a total of 26 (31%) sarcoidosis patients had eosinophilia. The frequency of mild PBE in sarcoidosis is very variable and reaches up to 25% [19]. In the study of Renston et al. on sarcoidosis patients, the PBE frequency was found to be 41% in sarcoidosis when the threshold value was 4% [27]. Another study by Renston et al. investigating 178 sarcoidosis patients and using the eosinophilia threshold value (>4%) found the frequency of eosinophilia to be 35.4%. In their study they explained that the presence of PBE is not only associated with the accompanying allergic disease (e.g., asthma, allergic rhinitis) and that eosinophilia can be directly associated with sarcoidosis [28]. In our study 26 (31%) patients had PBE, of these patients 6 had history of asthma. In addition, there was no significant difference between the sarcoidosis stage and the mean PBE. In the study by Renston et al., no significant difference was found between sarcoidosis stages showing similarity to this study. When these data were combined, as the sarcoidosis stage increased, there was no change in the number of eosinophils [27]. In the future, prospective studies may be needed in order to define if there is a relation between PBE and exacerbation/mortality in sarcoidosis patients.

As for the respiratory patterns 31 (36.9%) patients had obstructive, 12 (14.3%) restrictive and 4 (4.8%) mixed airway disorders. Reversibility was observed in 6 (7.1%) patients, four of whom were previously diagnosed with asthma. Although mostly normal PFT is seen in sarcoidosis, restrictive, obstructive or mixed disorder can also be observed. In a study done by Danila et al. only a minority of the patients had a reduction in lung volume and airflow obstruction [5]. As for this study, 36.9% of airflow obstruction were observed in sarcoidosis.

While normal respiratory function test is expected in most of the sarcoidosis patients, 44.04% normal PFT was observed in our study. The lower than expected rate was thought to be due to the absence of stage 0 patients which may be due to patients being asyptomatic at that stage and not applying to the hospital. Also another explanation would be related to the fact that this group of patients may be overlooked/underdiagnosed because of normal chest X-ray.

Since sarcoidosis primarily affects the lung structure, it is generally known as a restrictive lung disease. The restrictive breathing pattern is thought to be due to advanced fibrosis secondary to parenchymal sarcoid granuloma and/or concomitant interstitial pneumonia [14]. In a study, the frequency of restrictive and mixed respiratory dysfunction was found to be 14.6% in newly diagnosed sarcoidosis patients, while 9.7% obstructive breathing disorder was observed [29]. In another study, the frequency of restrictive airway disorder was found to be 20% [30]. The difference in the obstructive PFT ratio from other studies and this one may be due to the abscence of stage 0 patients, the nonhomogen female to male ratio and the extent of lung paranchyma and airway involvement.

One study was performed on 80 stages 1–3 sarcoidosis patients, and as the stage progressed, a significant decrease was observed in FEV1% and FVC%, but no significant decrease in FEV1/FVC ratio was noticed [19]. In another study, the evaluation of PFT values of 130 stages 1–4 sarcoidosis patients showed a significant decrease in FEV1/FVC, FEV1% and FVC%, respectively, as the stage progressed [31]. In this study, only FEV1 (milliliters and percentage) decreased significantly as the stage progressed. This significant decrease was thought to be due to endobronchial sarcoidosis granulomas causing endobronchial fibrosis and narrowing in the airways [30]. As for the absence of significant decrease in FEV1/FVC and FVC, it is thought to be due to the low number of stages 3 and 4 sarcoidosis patients.

In a study done upon 228 sarcoidosis patients no reversible PFT was observed [32]. However, a review of sarcoidosis showed that approximately 20% of patients have airway hyperreactivity [29]. It is recommended to use inhaled corticosteroids (ICS), especially in cough associated with sarcoidosis. It is thought that ICS has a suppressive effect on both airway hyperreactivity and granuloma in the airway [31]. About 7.1% of our patients had a reversible PFT and these patients may benifit from ICS but further studies may be needed. Out of the 14 patients with smoking history only 2 had an obstructive airway disorder. At the same time, 9 out of 31 patients with obstructive PFT had a history of asthma. Considering these results, it was understood that airway obstruction due to sarcoidosis alone can be observed in accordance with the literature [5].

Out of the 84 patients included in the study 6 (7.1%) had a reversible obstruction in PFT, 4 of whom had a self-reported asthma history.

The average DLCO of the patients included in the study was found to be 88.58%. Increased diffusioncapacity for carbon monoxide was observed in 6 patients. All of these patients had a history of asthma and it was concluded that this increase may be due to asthma. While most of the patients (58.3%) had normal DLCO values, 29 patients (34.5%) had low DLCO. Due to parenchymal involvement and fibrosis alveo-capillary membrane thickening in sarcoidosis, a decrease in DLCO is expected. DLCO is known as the most sensitive parameter to detect functional alveolar surface area loss. In two separate studies, as the sarcoidosis stage progressed, a significant decrease in DLCO values of 80 stages 1–3 sarcoidosis patients in the first and 130 stages 1–4 sarcoidosis patients in the second study were observedwhich is compatible with our findings [30,31].

Considering the results obtained from the survey, it is understood that obstructive airway disorder and reversible PFT can be seen in patients with sarcoidosis without history of asthma and smoking. Wheezing, chest tightness, shortness of breath and cough can be seen in both asthma and sarcoidosis. It was also observed that hay fever, eczema, skin and nose allergy may be seen both in asthma and sarcoidosis. Therefore, having very similar findings and symptoms between asthma and sarcoidosis can delay the diagnosis of sarcoidosis. As a result, we may be able to say that atopic diseases frequently accompany sarcoidosis. In such patients who are diagnosed with asthma and did not respond well to treatment, a lung tomography may be needed for futher evaluation.

## 5. Limitations

We had some limitations to our study such as not having a control group in which PBE could be compared. The stages of sarcoidosis patients were not homogenous in terms of patient numbers in each group and the general patient population was low. To evaluate atopy and allergy in more detail serum specific IgE and skin prick test could have been more helpful along with the survey. Although patients were included consecutively to the study the patient population was mostly women.

## 6. Conclusion

Mild PBE may be seen in sarcoidosis patients but it is independent from the disease stage. Patients applying with PBE, airway obstruction, bronchial hyperreactivity, hay fever, eczema, skin/nose allergy, wheezing, chest tightness, shortness of breath and cough whom are evaluated in terms of asthma but do not respond to treatment may need to be evaluated for sarcoidosis. Since the symptoms are not specific to sarcoidosis, it can sometimes be difficult to diagnose asthma in a patient who has previously been diagnosed with sarcoidosis.

## Figures and Tables

**Table 1:** Demographics, laboratory and pulmonary function tests.

Variable	Average ± SD
Sex M:F (%)	14 (16.7%):70 (83.3%)
Age (years)	48.39 ± 12.97
BMI (kg/m^2^)	30.74 ± 5.26
Hemoglobin (mg/dL)	13.55 ± 1.58
PBE absolute value (per μL)	254.04 ± 176.41
PBE%	3.49 ± 2.35
FEV1/FVC	77.04 ± 7.29
FEV1 (mL)	2403.83 ± 759.21
FEV1%	93.97 ± 18.98
FVC (mL)	3109.52 ± 880.58
FVC%	102.89 ± 18.33
Reversibility (mL)	119.52 ± 106.89
Reversibility%	5.01 ± 4.27
DLCO (mL/mmHg/min)	88.58 ± 19.66

**SD:** Standard deviation, **BMI:** Body mass index, **PBE:** Peripheral blood eosinophilia **FEV1:** Forced expiratory volume in the 1st second, **FVC:** Forced vital capacity, **DLCO:** Diffusing capacity.

**Table 2:** PBE, pulmonary function tests and diffusing capacity results according to stage of disease.

		Stage 1 (n = 26)	Stage 2 (n = 45)	Stage 3 (n = 8)	Stage 4 (n = 5)	p value*
PBE	Mean ± SD	280.76	232.66	215.00	370.00	0.286
		± 216.20	± 151.63	± 117.22	± 216.79	
FEV1/ FVC	Mean ± SD	79.1154	75.0667	81.2500	77.4000	0.272
		± 5.92	± 7.67	± 3.88	± 10.13	
FEV1 (mL/sn)	Mean ± SD	2778.15	2270.44	2273.75	1866.00	0.013
		± 878.70	± 619.36	± 882.20	± 216.17	
FEV1 (%)	Mean ± SD	101.80	90.55	92.12	87.00	0.041
		± 12.65	± 18.88	± 30.44	± 18.34	
FVC (mL)	Mean ± SD	1860.00	1540.00	1890.00	1720.00	0.147
		± 6160.00	± 5450.00	± 4400.00	± 2960.00	
FVC (%)	Mean ± SD	108.38	102.00	94.50	98.80	0.182
		± 13.83	± 17.73	± 28.13	± 22.92	
DLCO (%)	Mean ± SD	96.6538	87.9556	85.8750	56.6000	0.00
		± 16.62	± 16.77	± 28.07	± 10.16	

**PBE:** Peripheral blood eosinophilia, **SD:** Standard deviation, **FEV1:** Forced expiratory volume in the 1st second, **FVC:** Forced vital capacity, **DLCO:** Diffusing capacity,

* One-way ANOVA.

**Table 3:** Asthma/allergy survey.

Survey questions	Total patients		Male (n = 14)	Female (n = 70)	p value*
	N	%	N (%)	N (%)	
Have you ever been diagnosed by your doctor as having asthma?	24	28.6	3 (21.4)	21 (30)	>0.05
If yes—have you had an attack of asthma in the last 12 months?	6	7.1	2 (14.2)	4 (5.7)	>0.05
Have you ever taken medicine (including inhalers, aerosols or tablets) for asthma?	18	21.4	3 (21.4)	15 (21.4)	>0.05
If yes—are you currently taking any medicine (including inhalers, aerosols or tablets) for asthma?	13	15.5	3 (21.4)	10 (14.2)	>0.05
Have you had wheezing or whistling in your chest at any time in the last 12 months?	21	25.0	4 (28.5)	17 (24.2)	>0.05
If yes—have you been at all breathless when the wheezing noise was present?	15	17.9	2 (14.4)	13 (18.5)	>0.05
Have you had this whistling or wheezing when you did not have a cold?	10	11.9	1 (7.1)	9 (12.8)	>0.05
Have you woken up with a feeling of tightness in your chest at anytime in the last 12 months?	19	22.6	3 (21.4)	16 (22.8)	>0.05
Have you been woken by an attack of shortness of breath at any time in the last 12 months?	18	21.4	2 (14.4)	16 (22.8)	>0.05
Have you been woken by an attack of coughing at any time in the last 12 months?	17	20.2	4 (28.5)	13 (18.5)	>0.05
Do you have allergies such as hay fever, eczema, skin or nose allergy?	17	20.2	2 (14.2)	15 (21.4)	>0.05
Did/does your mother have asthma?	9	10.7	0 (0)	9 (12.8)	>0.05
Did/does your mother have any allergies such as hay fever, eczema, skin or nose allergy?	1	1.2	0 (0)	1 (1.4)	>0.05
Did/does your father have asthma?	8	9.5	1 (7.1)	7 (10)	>0.05
Did/does your father have any allergies such as hay fever, eczema, skin or nose allergy?	1	1.2	0 (0)	1 (1.4)	>0.05
Do any of your brothers and sisters have asthma?	7	8.3	0 (0)	7 (10)	>0.05
Do any of your brothers and sisters have any allergies such as hay fever, eczema, skin or nose allergy?	2	2.4	0 (0)	2 (2.8)	>0.05
Have you ever smoked for as long as a year?	14	16.7	3 (21.4)	11 (15.7)	>0.05
Do you smoke now as of one month ago?	4	4.8	1 (7.1)	3 (4.2)	>0.05

* Chi-square test.
